# Supplementary material for: Distance- and Hierarchy-Dependent Functional Dysconnectivity in Schizophrenia and Its Association with Cortical Microstructure
Source: medRxiv. 2025 Jul 28:2025.07.28.25332321. Preprint. [Version 1] doi: 10.1101/2025.07.28.25332321 (PMC12324661; doi:10.1101/2025.07.28.25332321)
Supplement: 1 [file NIHPP2025.07.28.25332321V1-supplement-1.pdf]

medRxiv preprint doi: https://doi.org/10.1101/2025.07.28.25332321; this version posted July 28, 2025. The copyright holder for this preprint (which was not certified by peer review) is the author/funder, who has granted medRxiv a license to display the preprint in perpetuity.
It is made available under a CC-BY 4.0 International license .

Supplement

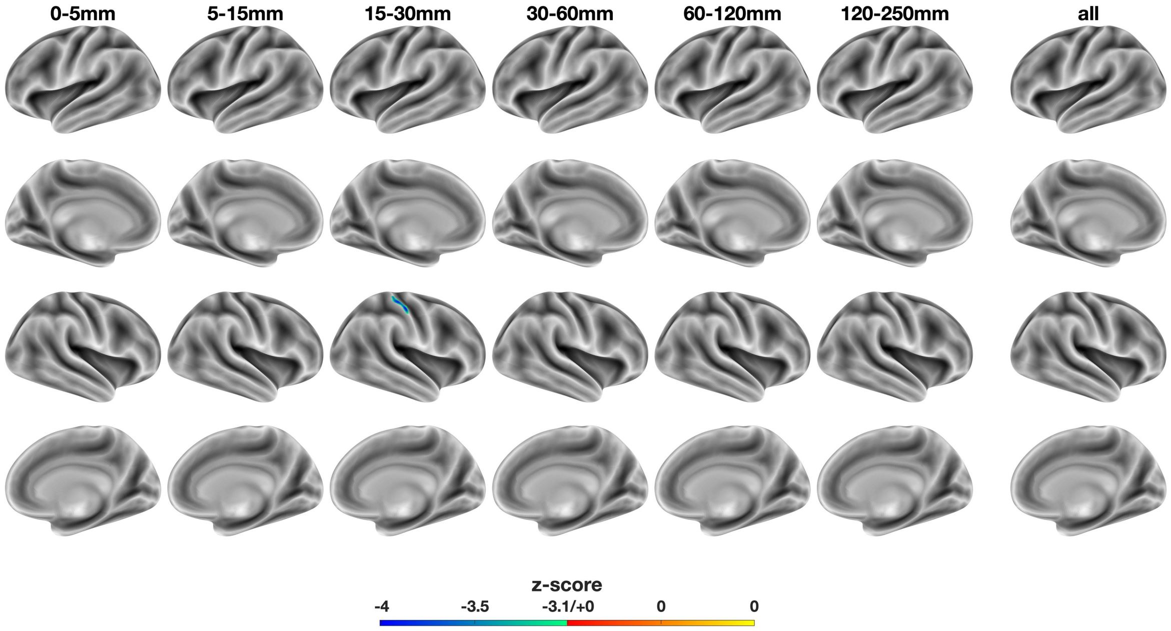

Figure S1. Voxel-wise, distance-dependent differences in absolute functional connectivity strength (aFCS): SSD - HC. *The colorbar represents z-score, with warm and cold colors indicating increased and decreased aFCS in SSD, respectively. Column labels denote distance ranges used to compute voxel-wise aFCS. The rightmost "all" column shows significant differences in global aFCS (i.e. non-distance-dependent). SSD: schizophrenia spectrum disorder. HC: healthy controls. Only the right dorsal S1 cluster at 15-30 mm remains significant in the aFCS analysis.*

medRxiv preprint doi: https://doi.org/10.1101/2025.07.28.25332321; this version posted July 28, 2025. The copyright holder for this preprint (which was not certified by peer review) is the author/funder, who has granted medRxiv a license to display the preprint in perpetuity.
It is made available under a CC-BY 4.0 International license .

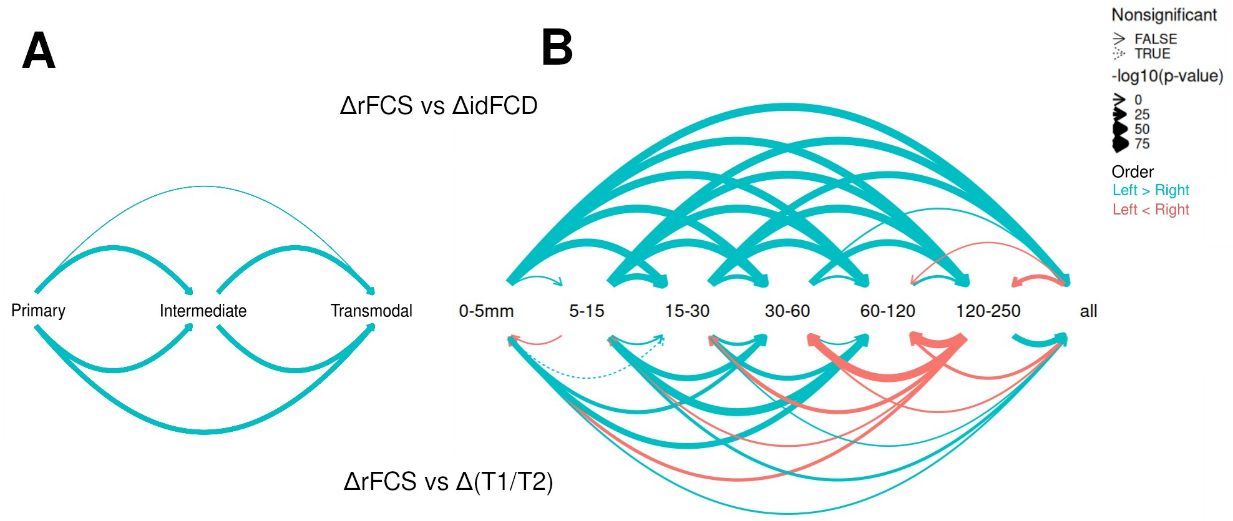

Figure S2. Statistical comparisons between correlations in functional and inferred structural alterations in SSD compared to HCs (continued from Fig 4B). *Remaining post-hoc pairwise comparisons among groups of correlations of SSD-HC differences in functional and inferred structural measures.* ***(A)*** *Comparisons among levels when grouping by network hierarchy alone.* ***(B)*** *Comparisons among levels when grouping by distance alone. In both cases, graph edges in the upper half represent comparisons of correlations between ΔrFCS and ΔidFCD. Lower half represents ΔrFCS and Δ(T1/T2). The direction of the effect (group with higher mean cross-product deviations) is given by arrow direction and color, with cyan standing for stronger correlations to the left of the plot. Smaller p-values (FWE-corrected) translate into thicker arrows. In general, primary networks display stronger correlations between functional and inferred structural alterations, which in turn display stronger correlations than transmodal networks. Similarly, correlations become weaker as more distant alterations in rFCS are considered.*

medRxiv preprint doi: https://doi.org/10.1101/2025.07.28.25332321; this version posted July 28, 2025. The copyright holder for this preprint (which was not certified by peer review) is the author/funder, who has granted medRxiv a license to display the preprint in perpetuity.
It is made available under a CC-BY 4.0 International license .

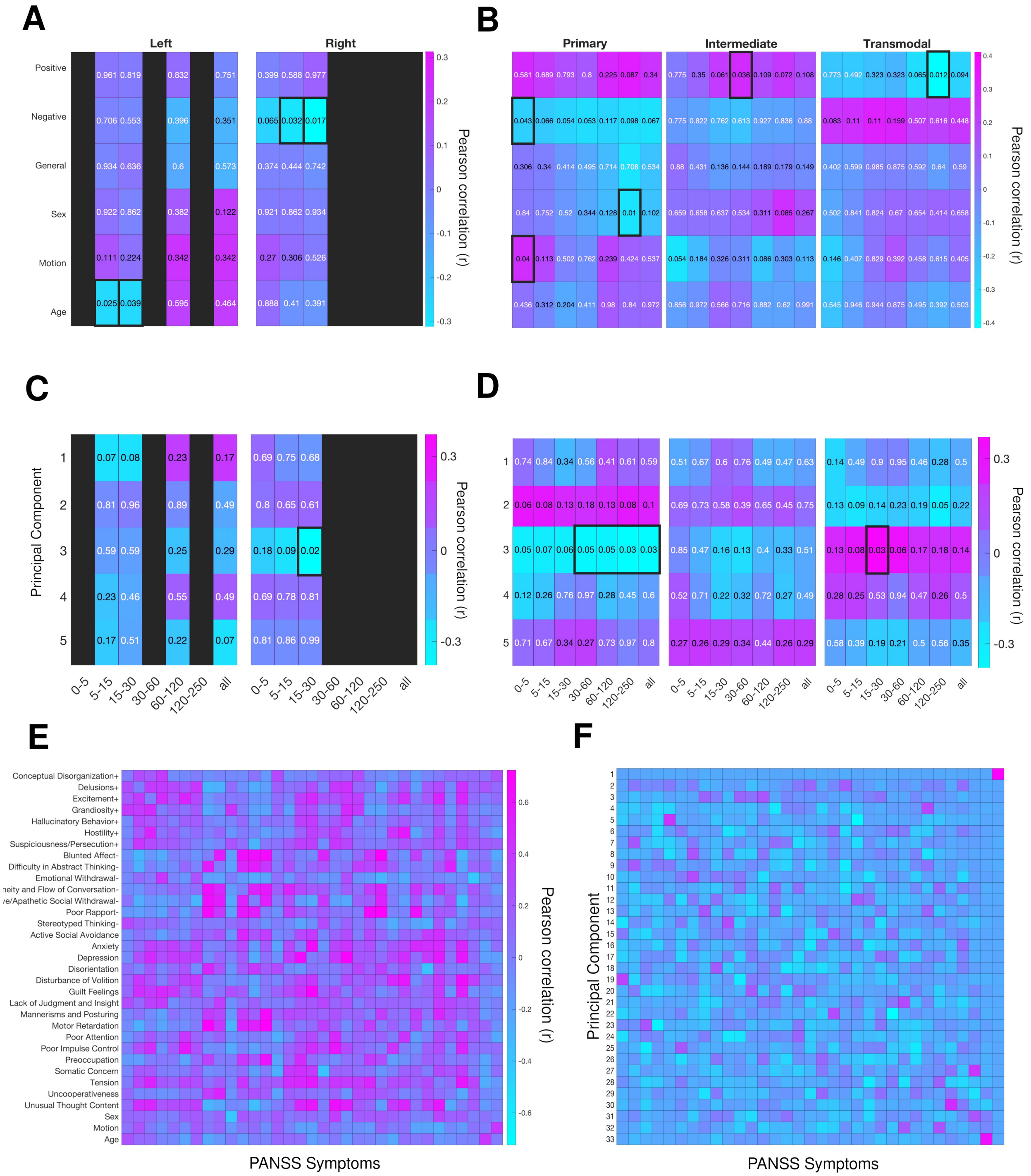

medRxiv preprint doi: https://doi.org/10.1101/2025.07.28.25332321; this version posted July 28, 2025. The copyright holder for this preprint (which was not certified by peer review) is the author/funder, who has granted medRxiv a license to display the preprint in perpetuity.
It is made available under a CC-BY 4.0 International license .

Figure S3. Correlation between rFCS and psychiatric symptoms in the COBRE dataset. ***(A)*** *Pearson correlations of PANSS subscale scores (positive, negative, general, plus confound variables) against individual-level average rFCS from significant clusters in Fig. 1. Number at each cell is the (uncorrected) p-value corresponding to the parameter for that symptom, according to a multiple linear regression fit of rFCS. Colorbar represents Pearson correlation, with pink for positive and blue for negative associations, respectively.* ***(B)*** *Same as (A) but correlating against average rFCS from hierarchy levels (primary, intermediate, and transmodal networks).* ***(C)*** *Same as (A) but using the principal components from (F) (only first 5 PC shown here), instead of original PANSS subscale scores and confounds.* ***(D)*** *Same as (C) but correlating against average rFCS from hierarchy levels (primary, intermediate, and transmodal networks).* ***(E)*** *Correlation matrix across patient scores for all 30 symptoms in the Positive and Negative Syndrome Scale (PANSS).* ***(F)*** *Projection of original PANSS scores on the components found by PCA*.
